# Supplementary material for: Mechanisms governing the pioneering and redistribution capabilities of the non-classical pioneer PU.1
Source: Nat Commun. 2020 Jan 21;11:402. doi: 10.1038/s41467-019-13960-2 (PMC6972792; doi:10.1038/s41467-019-13960-2)
Supplement: Supplementary file 7 — Source data [file 41467_2019_13960_MOESM7_ESM.zip › Source_Data/FigureS2/FigureS2B_MotifScanOutput/KmeansCluster7/homerResults/motif38.similar.html]

motif38

## Information for motif38

A
G
C
T
A
G
T
C
C
G
T
A
A
C
G
T
A
C
T
G
C
T
A
G
A
C
T
G
C
T
A
G
A
T
C
G
G
C
T
A
C
T
G
A
  
Reverse Opposite:  

A
G
C
T
C
G
A
T
T
A
G
C
G
A
T
C
T
G
A
C
A
G
T
C
A
G
T
C
G
T
C
A
A
C
G
T
A
C
T
G
C
T
G
A
  

|  |  |
| --- | --- |
| p-value: | 1e-33 |
| log p-value: | -7.690e+01 |
| Information Content per bp: | 1.717 |
| Number of Target Sequences with motif | 176.0 |
| Percentage of Target Sequences with motif | 5.48% |
| Number of Background Sequences with motif | 773.0 |
| Percentage of Background Sequences with motif | 1.77% |
| Average Position of motif in Targets | 97.3 +/- 64.3bp |
| Average Position of motif in Background | 105.0 +/- 69.2bp |
| Strand Bias (log2 ratio + to - strand density) | 0.4 |
| Multiplicity (# of sites on avg that occur together) | 1.15 |
| Motif File: | file (matrix) reverse opposite |

### Similar de novo motifs found

|  |  |  |  |  |  |  |  |
| --- | --- | --- | --- | --- | --- | --- | --- |
| Rank | Match Score | Redundant Motif | P-value | log P-value | % of Targets | % of Background | Motif file |
| 1 | 0.802 | T A G C A G C T A G T C C G T A A C G T A C T G C G T A | 1e-16 | -38.976041 | 10.16% | 6.08% | motif file (matrix) |
